# Turning canopy temperature into a multidimensional tool: phenotyping diurnal canopy temperature dynamics in spring wheat breeding programs

**DOI:** 10.3389/fpls.2026.1936928

**Published:** 2026-10-05

**Authors:** Hamza-Ali Khan, Iván Matus, Dalma Castillo, Josefa Pacheco, Alejandro del Pozo, Gustavo A. Lobos

**Affiliations:** 1 Plant Breeding and Phenomics Center, Faculty of Agricultural Sciences, Universidad de Talca, Talca, Chile; 2 CRI-Quilamapu, Instituto de Investigaciones Agropecuarias, Chillán, Chile

**Keywords:** curve fitting, diurnal variation, K-Means clustering, Mediterranean environment, stomatal conductance, thermometry, yield components

## Abstract

**Introduction:**

Canopy temperature (Tc) measured at 14:00 h is a standard procedure in wheat breeding programs for assessing crop water status and heat stress tolerance. However, a single reading captures only a fraction of the thermal variation that unfolds over a full day.

**Methods:**

This study phenotyped 196 spring wheat genotypes at two contrasting Mediterranean sites in Chile, a dryland rainfed site (WS) and a fully irrigated site (FI), during the 2024 growing season. Hourly Tc and canopy–air temperature differential (CTD) were recorded from 09:00 to 18:00 h across four phenological stages, including anthesis, milk stage, early grain filling, and late grain filling. Spatially corrected best linear unbiased estimates (BLUEs) were derived using Spatial Analysis of Field Trials with Splines (SpATS), and the best-fitting diurnal curve per genotype per stage was selected from ten candidate model families by minimizing the Bayesian Information Criterion (BIC). Curve parameters and pairwise rates of change (ROC) extracted from the fitted Tc and CTD profiles were evaluated as predictors of seven agronomic traits, including grain yield, harvest index, thousand-kernel weight, kernels per spike, kernels per square meter, shoot dry weight, and spikelets per square meter. Models were evaluated using in-sample partial least squares (PLS) regression R², nested cross-validated Q², a permutation-based null model, and hold-out-genotype testing.

**Results:**

The most predictive single measurement hour shifted across traits, stages, and sites with no consistent pattern, and the conventional 14:00 h reading was the best single hour in about 9% of stage-trait combinations tested. When all features within each predictor category were used jointly in a PLS regression, in-sample R² exceeded the 14:00 h control across most site–stage combinations, and k-means clustering further increased within-cluster R², though these apparent gains may be inflated by predictor number and cluster subsetting. Under stricter, cross-validated evaluation, whole-panel Q² was close to zero for all predictor categories (median -0.023 to -0.009), no cluster consistently outperformed random genotype partitions on held-out genotypes, and representative cluster-level Q² values ranged from -0.31 to 0.76.

**Discussion:**

These results indicate that in-sample R² substantially overestimated the predictive value of diurnal clustering, and that cross-validated Q², not in-sample R², should be the primary metric for such claims.

## 1 Introduction

Heat stress during reproductive development poses a primary constraint on global wheat production. Climate projections anticipate an increase of approximately 2 °C–3 °C in temperatures by mid-century (Carvalho et al., 2021). Yield losses of approximately 6% per degree Celsius were projected for major wheat-producing regions (Asseng et al., 2015). The pressure is particularly acute in Mediterranean climatic zones, where drought and heat stress regularly cause large yield losses (del Pozo et al., 2020). In the central regions of Chile, mean growing season temperatures are projected to increase by 1.5 °C by mid-century (González and Velasco, 2008), while precipitation variability has intensified, compressing the window of favorable conditions between anthesis and physiological maturity (del Pozo et al., 2022). Developing heat-tolerant cultivars therefore demands reliable, scalable phenotypic indicators for large genotype panels.

Canopy temperature (*T*_c_) measured with handheld or unmanned aerial vehicle (UAV)-mounted infrared thermometers has long been used as a proxy for evaporative cooling capacity and crop water status (Al-Najadi et al., 2025; Bates et al., 2025; Jackson et al., 1981). When *T*_c_ drops below air temperature (*T*_a_), the canopy–air temperature differential (CTD) is negative, indicating active transpirational cooling from open stomata. A positive CTD marks a canopy warmer than its surroundings and is associated with stomatal closure under heat or water stress (Fukai and Mitchell, 2022). Both *T*_c_ and CTD have been linked to grain yield in water-limited environments and used as selection criteria in CIMMYT breeding programs (Nehe et al., 2021). However, canopy temperature patterns are not static within the day and across developmental stages. Previous work has shown that thermal profiles vary substantially across grain-filling stages, with “old-type” and “warm-type” genotypes diverging during the middle to late stages of grain filling (Yu et al., 2025).

Despite its physiological relevance, *T*_c_ has almost always been measured at a single afternoon time point, typically between 12:00 and 15:00 h (Ohnishi et al., 2021). The 14:00 measurement is used as the optimal time for monitoring stress levels (Wang et al., 2024), when the canopy–air difference is at its daily peak (Rutkoski et al., 2016). This convention reduces workload in large trials but discards information encoded in the full diurnal trajectory. The question of when to measure *T*_c_ is inseparable from the question of what thermal property matters most for yield. Does grain yield correlate best with peak canopy temperature, the timing of maximum cooling, integrated thermal load, heating rate, evening recovery, or thermal hysteresis between morning and afternoon trajectories? Each of these quantities can only be estimated from a time-resolved diurnal record. The canopy warms in the morning as radiation rises, peaks in early afternoon, then cools as stomata respond to decreasing radiation and increasing vapor pressure deficit (VPD) (Balota et al., 2007). Genotypes differ not only in peak temperature but also in heating rate, timing of thermal maximum, plateau width, and recovery of cooling capacity in the afternoon. These dynamic features reflect underlying stomatal kinetics and transpiration efficiency, traits directly relevant to stress tolerance and yield stability (Schoppach and Sadok, 2012; Thapa et al., 2018).

A complementary dimension of this problem concerns genotypic stratification. Wheat germplasm panels often contain genotypes with fundamentally different thermo-hydraulic strategies (Moritz et al., 2024). High-transpiration-type plants maintain cool canopies throughout the day by accessing deep soil water reserves. Conservative types close their stomata to limit water loss and have warmer afternoon canopies. This allows soil moisture retention for late grain filling (Thapa et al., 2018). These strategies may produce similar yields under mild stress but diverge sharply under severe or prolonged drought (Schoppach and Sadok, 2012). If phenotypic clustering is performed using only a single *T*_c_ observation, both strategies may map to the same thermal class at that hour, obscuring a biologically meaningful distinction. A diurnal thermal profile, by contrast, encodes the signature of each strategy across the full atmospheric demand gradient of the day, enabling biologically interpretable cluster-based stratification for breeding decisions.

Methodological advances now make systematic diurnal *T*_c_ phenotyping at scale practical. High-throughput infrared thermometry, handheld or UAV-mounted, can capture hourly canopy temperature across hundreds of plots within a single day (Flores-Olave et al., 2026). Diurnal *T*_c_ and CTD trajectories yield a range of features, including curve parameters, timing metrics, and pairwise rates of change (ROC). Whether these features improve prediction of agronomic traits compared with single-time-point measurements remains untested.

Studies in rice and maize have used diurnal temperature profiles and remote sensing time series to capture crop physiological dynamics beyond point-in-time measurements (Rossini et al., 2015; Wu et al., 2023). In wheat, Thapa et al. (2018) showed that the severity of grain-filling impairment depends on both the magnitude and timing of heat exposure. Reductions in the grain-filling period often outweigh compensatory increases in grain-filling rate. Despite the documented diurnal variation in canopy temperature, no study has yet systematically extracted and validated the full suite of diurnal *T*_c_ and CTD curve features as predictors of yield-related traits across a large spring wheat panel in contrasting environments.

Three objectives guided this study: (1) to determine whether diurnal *T*_c_ and CTD features predict agronomic trait variation more effectively than the conventional 14:00 h single measurement across stages and sites; (2) to characterize the diversity of best-fitting diurnal curve shapes within the panel using Bayesian Information Criterion (BIC)-based model selection; and (3) to evaluate whether *k*-means clustering on the thermal feature space reveals genotypic subgroups with improved within-group trait predictability.

## 2 Materials and methods

### 2.1 Plant material and field design

A panel of 196 advanced lines and cultivars of spring bread wheat (*Triticum aestivum* L.), exhibiting similar phenology and previously characterized variability under rainfed conditions (WS), was selected from a larger panel of 384 genotypes (del Pozo et al., 2016). This panel was evaluated at two sites during the 2024 Chilean growing season, Cauquenes (35°58′S, 72°17′W; 177 m.a.s.l.; Mediterranean dryland, ~ 500 mm annual rainfall − WS) and Santa Rosa (36°32′S, 71°55′W; 220 m.a.s.l.; irrigated, 680 mm supplemental irrigation – FI). The two sites represented contrasting thermal regimes. Cauquenes experienced higher mean maximum temperatures during grain filling (up to 38 °C recorded at MS), whereas Santa Rosa was more temperate, with peak canopy temperatures generally 3 °C–4 °C cooler during the second grain filling stage (LGF).

At each site, the panel was planted in an alpha-lattice incomplete block design with two replicates. Plots comprised five rows, each 2.0 m long, spaced 0.20 m apart, giving a total plot area of 2.0 m^2^ (2.0 m × 1.0 m). Sowing rate was 20 g m^−2^.

### 2.2 Phenological stage classification and agronomic trait measurements

Phenological development was tracked on a plot-by-plot basis using the Zadoks decimal growth stage (GS) scoring every 3 days (Zadoks et al., 1974). Measurement days were selected when the majority of a plot had reached the target growth stage. The four target growth stages were anthesis (ANT, GS 61–65), milk stage (MS, GS 73–75), early grain filling (EGF, GS 83–85), and late grain filling (LGF, GS 87–89).

At physiological maturity, plots were mechanically harvested, and grain yield (GY) was recorded on a per-plot basis and converted to tons per hectare. Subsamples were used to determine thousand-kernel weight (TKW; g per 1,000 kernels counted on a seed counter), kernels per spike (KS), kernels per square meter (KM2), and harvest index (HI = GY/shoot dry weight). Shoot dry weight (SDW; g m^−2^) was determined by drying aboveground biomass at 70 °C for 72 h. Spikelets per square meter (SM2) were estimated from spike counts per square meter.

### 2.3 Canopy temperature measurements

For each developmental stage (ANT, MS, EGF, and LGF) and water treatment (fully irrigated [FI]; water-stressed [WS]), the diurnal course of *T*_c_ was assessed through 10 thermal imaging flights conducted at hourly intervals from 09:00 to 18:00 h, with measurements taken on a single day per stage.

A DJI 200 V2 UAV (Dajiang Innovation Technology Co. Ltd., Shenzhen, China) equipped with a dual-camera Zenmuse XT2 (FLIR, Wilsonville, OR, USA) was employed for data acquisition. The following parameters were standardized, including (a) flight altitude of 30 m, (b) image overlap of 85% in both the forward and side directions, (c) flight speed of 2.3 m s^−1^, and (d) flights conducted only under cloudless, sunny conditions with wind speeds not exceeding 15 km h^−1^. The camera was activated at 1.5-s intervals via an intervalometer. Prior to the commencement of each mission, the thermal cameras were activated to ensure the thermal equilibrium of the internal sensor components (Kelly et al., 2019).

During image collection, the emissivity value was initially set to 1; however, for subsequent processing, it was adjusted to 0.96 for vegetated surfaces (Jones, 2004; Leinonen et al., 2006) while remaining at 1 for reference targets. A field-based empirical linear radiometric calibration of the UAV-acquired temperature data was carried out following the protocol established by Gómez-Candón et al. (2016). For each flight, ground reference temperatures were recorded from various artificial targets (white and gray fabrics and a water-filled container) using a FLUKE 62 MAX IR thermometer (Fluke Corporation, Everett, WA, USA). Furthermore, nine square ground control points (0.5 m × 0.5 m, black-and-white patterned) were strategically positioned across the field and georeferenced with a Differential Global Positioning System (DGPS) (GeoXT Handheld Trimble, Houston, TX, USA). These reference points, in conjunction with the UAV’s onboard GPS data, were subsequently employed to enhance the accuracy of orthomosaic geolocation.

The raw digital numbers (DN) from each thermal image were converted to Celsius temperature using a custom FIJI script. Orthomosaics were then generated for each flight in Agisoft PhotoScan Professional 2.0 using a structure-from-motion (SfM) algorithm (Triggs et al., 2000), following the workflow of Perich et al. (2020). The exported orthomosaics were then processed with interactive filtering in custom MATLAB R2025a code to remove nonvegetative material (Fuentes et al., 2012). The MosaicTools plugin in FIJI was then used to identify individual genotype plots and extract *T*_c_. Plot-level extraction used the Otsu thresholding algorithm (Otsu, 1979), which automatically separates soil and vegetation pixels based on a bimodal intensity distribution (Santini et al., 2019). The mean *T*_c_ per plot and replicate was subsequently computed using vegetation-classified pixels. This process was automated through a custom FIJI batch-processing macro. Air temperature (*T*_a_) was recorded from a weather station located adjacent to the experimental field. CTD was calculated as the difference between *T*_c_ and *T*_a_ (Hagan Brown et al., 2025; Martínez et al., 2017) as described in Equation 1.

(1) $$\text{CTD}\;=\;T_{c}-T_{a}$$

### 2.4 Data analysis

The analysis aimed to compare the associations between diurnal thermal features and agronomic traits, and to evaluate whether genotypic subgroups exhibited stronger trait associations than the entire population. Seven predictor categories were derived from the hourly BLUEs and compared against the conventional 14:00 h *T*_c_ reading as the control. The analysis proceeded in four steps. First, each individual hourly *T*_c_ reading was compared against the control to determine whether the most predictive measurement time was consistent across stages and sites. Second, all features within each category were used jointly in a partial least squares (PLS) regression across all 196 genotypes without clustering, establishing a multivariate baseline for each category. Third, *k*-means clustering was applied to each feature category to identify genotypic subgroups with improved within-group trait predictability. Fourth, in-sample *R*^2^ can increase with the number of predictors and may be inflated when group size approaches the number of predictors. Therefore, all *R*^2^ comparisons across predictor categories and clusters were reevaluated using nested cross-validated *Q*^2^, a permutation-based null model, and hold-out-genotype validation. These validation procedures are described below.

We specify whether differences in *R*^2^ and *Q*^2^ across predictor categories, clusters, and measurement hours represent formally tested effects or descriptive patterns. Hourly and predictor-category comparisons (steps 1 and 2) are descriptive. In contrast, *k*-means clustering comparisons (step 3) were formally evaluated against a permutation-based null model, as described in the **Cross-validated model assessment** section.

#### 2.4.1 Spatial correction and BLUEs estimation

Field spatial heterogeneity was corrected using the Spatial Analysis of Field Trials with Splines (SpATS) algorithm (Rodríguez-Álvarez et al., 2018) as implemented in the R package statgenSTA. For each combination of environment (WS and FI), phenological stage (ANT, MS, EGF, and LGF), and daily measurement time (10 times per day), a two-dimensional penalized spline surface (design = “res.rowcol”, spatial = TRUE) was fitted jointly with the alpha-lattice incomplete block design. This was used to estimate Best Linear Unbiased Estimators (BLUEs) per genotype, using the fitTD() function in statgenSTA. No genotypes were excluded due to missing measurements, and all 196 genotypes were retained across all environment–stage combinations. This spatial correction approach has been successfully applied in UAV-based thermal phenotyping studies to improve genotypic discrimination under variable field conditions (Treier et al., 2025).

All analyses were performed in R version 4.6.1 (2026-06-24) using the statgenSTA (version 1.0.15), pls (version 2.9.0), mgcv (version 1.9.4), and tidyverse (version 2.0.0) packages. *k*-means clustering was performed using the base R stats::kmeans() implementation with the Hartigan–Wong algorithm.

#### 2.4.2 Diurnal curve fitting and BIC-based model selection

The 10 hourly BLUE values (9:00–18:00 h) for each genotype and phenological stage were used to construct the diurnal profiles of *T*_c_ and CTD. Ten candidate model families were included to span the full range of diurnal curve shapes plausible under field conditions. The parametric models include the symmetric single-peaked functions (Gaussian, Lorentzian, Sine), asymmetric functions (Gompertz, Exponential Rise-Fall), flexible polynomial families (Quadratic, Cubic), a threshold-response function (Piecewise Linear), and a fully nonparametric reference generalized additive model (GAM). This extent was necessary because no single temperature function adequately describes all genotypes across diverse stages and environments (van Voorn et al., 2023). The best-fitting model per genotype and stage was selected by minimizing the BIC (as in Equation 2), which penalizes model complexity in proportion to the number of parameters and is appropriate for comparing nonnested models (Schwarz, 1978). Each candidate model was fitted to the ten hourly observations available per genotype. All model fitting was performed in RStudio using nonlinear least-squares (nls) for parametric families and the mgcv package for GAM.

(2) $$\text{BIC}\;=\;k\text{ln}(n)-2\text{ln}(\hat{L})$$

where *k* is the number of model parameters, *n* is the number of observations (here *n* = 10 hourly readings), and $\hat{L}$ is the maximized likelihood of the fitted model.

From the best-fit model, 10 *T*_c_ and 10 CTD curve parameters were extracted (**Table 1**; **Figure 1**).

**Table 1:** Diurnal thermal features extracted from fitted canopy temperature (*T*_c_) and canopy–air temperature differential (CTD) curves for each genotype, stage, and site combination.

No.	Variable	Parameter	Description
1	*T*_c_	Tc_max	Maximum fitted canopy temperature (°C)
2	*T*_c_	Tc_min_9am	Fitted temperature at 09:00 h (°C)
3	*T*_c_	Tc_peak_time	Time of day at which fitted *T*_c_ reaches its maximum (decimal hours)
4	*T*_c_	Tc_AUC	Area under the fitted diurnal *T*_c_ curve from 09:00 to 18:00 h (°C·h^−1^)
5	*T*_c_	Tc_FWHM	Full width at half maximum of the diurnal thermal peak (h)
6	*T*_c_	Tc_Max_Heating_Rate	Maximum instantaneous rate of *T*_c_ increase during the morning phase (°C h^−1^)
7	*T*_c_	Tc_Max_Cooling_Rate	Maximum instantaneous rate of *T*_c_ decreases during the afternoon phase (°C h^−1^)
8	*T*_c_	Tc_Inflection_Point	Time of day at which the morning heating rate is greatest (decimal hours)
9	*T*_c_	Tc_Thermal_Hysteresis	Difference in fitted *T*_c_ between the morning and afternoon branches at equivalent temperatures (°C)
10	*T*_c_	Tc_Thermal_Onset_Time	Time at which fitted *T*_c_ first exceeds 30 °C (Ullah et al., 2022)
11	CTD	CTD_min	Most negative fitted CTD value, indicating maximum canopy warming relative to air (°C)
12	CTD	CTD_min_time	Time of day at which fitted CTD reaches its minimum value (decimal hours)
13	CTD	CTD_AUC	Area under the fitted diurnal CTD curve from 09:00 to 18:00 h representing cumulative cooling load (°C·h)
14	CTD	CTD_Range	Difference between maximum and minimum fitted CTD values across the diurnal cycle (°C)
15	CTD	CTD_FWHM_Cooling	Full width at half maximum of the CTD cooling phase, indicating duration of active transpirational cooling (h)
16	CTD	CTD_Midday	The fitted CTD value at 12:00 h, representing the baseline canopy cooling capacity (°C)
17	CTD	CTD_Terminal	The fitted CTD value at 18:00 h, representing the residual canopy cooling capacity (°C)
18	CTD	CTD_Morning_Change_Rate	Rate of CTD change during the morning phase from 09:00 h to peak cooling (°C h^−1^)
19	CTD	CTD_Evening_Recovery_Rate	Rate of CTD change during the evening phase from peak cooling toward 18:00 h (°C h^−1^)
20	CTD	CTD_Max_Cooling_Capacity	Maximum fitted CTD value, indicating the greatest canopy cooling below air temperature (°C)

**Figure 1:**
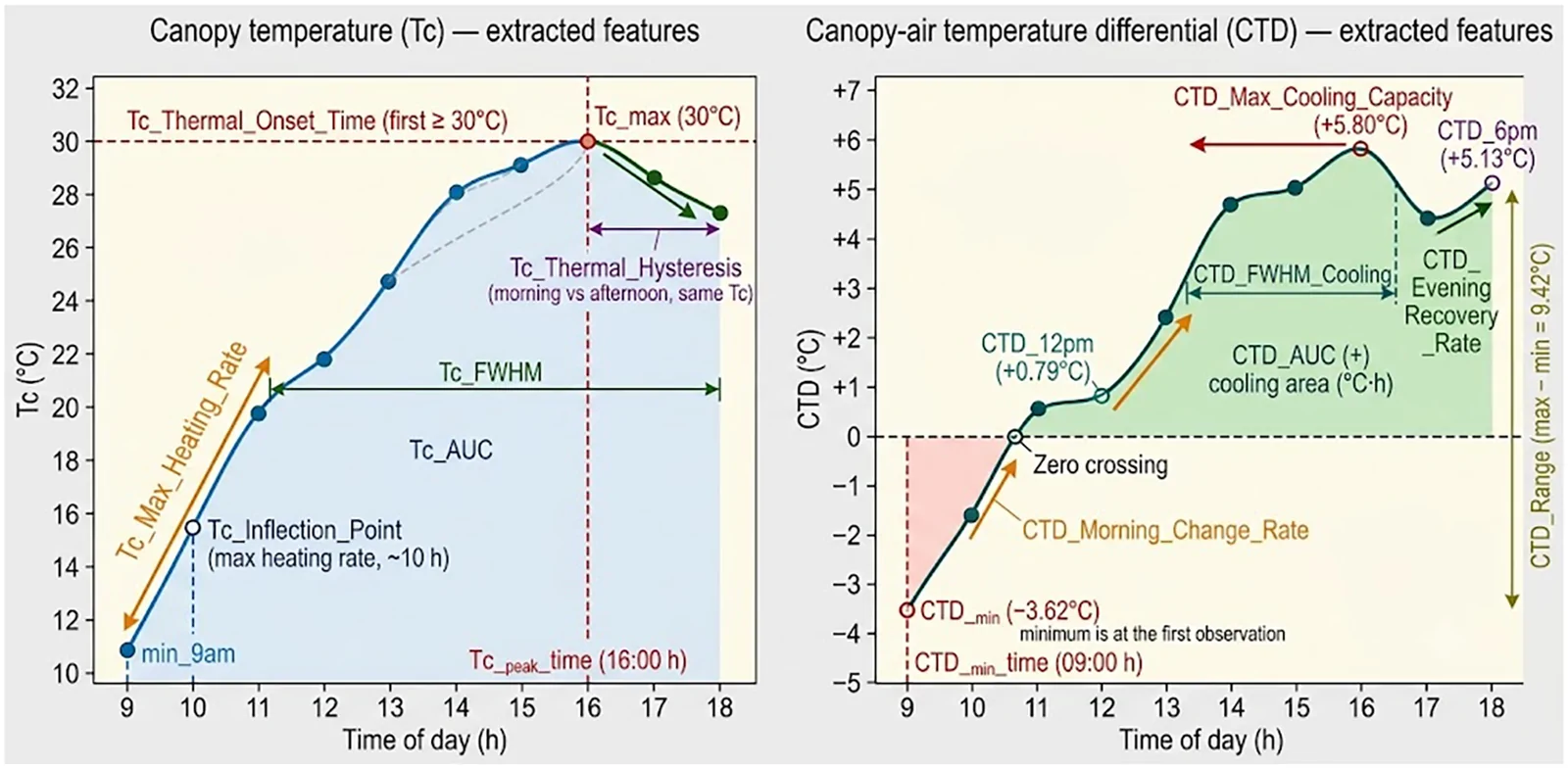
Conceptual diagram of diurnal thermal features. Features are extracted from canopy temperature (*T*_c_; left) and canopy–air temperature differential (CTD; right) curves. Each labeled point, arrow, or shaded segment corresponds to a specific phenotypic trait computed from the best-fit model. *T*_c_ and CTD features are explained in **Table 2**.

#### 2.4.3 Predictor categories

Six categories of diurnal thermal features were derived from the spatially corrected hourly BLUEs of *T*_c_ and CTD. Raw hourly *T*_c_ (All_10_Hours_TC) comprised the 10 observed canopy temperature readings from 09:00 to 18:00 h, capturing the absolute thermal level at each measurement time. Raw hourly CTD (All_10_Hours_CTD) comprised the corresponding 10 canopy–air temperature differentials, encoding the transpirational cooling status at each hour. Curve parameters for *T*_c_ (CurveParams_TC) consisted of 10 features derived from the best-fit diurnal model per genotype (**Table 1**). Curve parameters for CTD (CurveParams_CTD) comprised 10 analogous parameters from the fitted CTD profile (**Table 1**). Pairwise ROC for *T*_c_ (ROC_TC) represents pairwise combinations of the 10 hourly readings across the diurnal cycle. Pairwise ROC for CTD (ROC_CTD) represents pairwise combinations of the 10 hourly CTD values. A seventh predictor set, All_Categories, concatenated all six feature categories into a single 130-predictor feature vector, representing the complete diurnal thermal phenotype of each genotype at a given stage and site.

#### 2.4.4 k-means clustering

In order to assess whether the diurnal thermal features could stratify the panel into genotypic subgroups with improved within-group trait predictability, *k*-means clustering was applied at *k* = 3 using scaled (zero-mean, unit-variance) feature vectors. Clustering was performed separately for each of the seven predictor categories plus the 14:00 h control, and separately for each water regime (WS, FI). The clustering was also evaluated at *k* = 2 and *k* = 4. However, *k* = 3 was selected because *k* = 4 produced clusters smaller than *n* = 10 genotypes for several high-dimensional feature categories. These categories were insufficient for stable PLS modeling, since component selection becomes unreliable as cluster size approaches the number of predictors (Wold et al., 2001). Furthermore, *k* = 2 provided insufficient resolution to distinguish more than two broad thermal strategies. The *k*-means function in *R* with the Hartigan–Wong algorithm was used to conduct all clustering.

For each clustering solution, trait–feature associations within each cluster were quantified using PLS regression (plsr function, pls package). The number of latent components was selected by leave-one-out cross-validation and capped at min (*n* = − 2, *p* = 10), where *n* and *p* are the cluster size and the number of predictors, respectively. PLS was used because several predictor categories contain more variables than genotypes within a cluster. Within-cluster *R*^2^ was computed (Equation 3) on the training set using the optimal number of components and was compared against the unpooled No_Clustering baseline fitted on the same feature set across all 196 genotypes.

(3) $$R^{2}=1-\frac{\sum_{i=1}^{n}{\left(y_{i}\;-\;{\hat{y}}_{i}\right)}^{2}}{\sum_{i=1}^{n}{\left(y_{i}\;-\;\bar{y}\right)}^{2}}$$

where $y_{i}$ is the observed trait value for genotype *i*, ${\hat{y}}_{i}$ is the value predicted by the PLS model using the optimal number of components, $\bar{y\;}$ is the mean observed trait value, and *n* is the number of genotypes in the cluster (or in the full panel for the no-clustering baseline).

In clusters where *n* approaches *p*, within-cluster *R*^2^ values represent upper bounds on predictability rather than generalizable estimates. This limitation motivated the cross-validated assessment described in the following section.

#### 2.4.5 Cross-validated model assessment

In-sample *R*^2^ can be inflated by both the number of predictors and the use of small clusters. Therefore, all *R*^2^ comparisons were reevaluated using three complementary out-of-sample procedures. These included the whole-panel PLS models without clustering and the within-cluster models.

For each combination of predictor category, trait, site, stage, and cluster, or the unclustered whole panel, an out-of-sample cross-validated loop was used to hold out data that were not available to the model-selection step. For groups with *n* ≤ 30 genotypes, full leave-one-out (LOO) cross-validation was used. For groups with *n* > 30, 10 repeats of 10-fold cross-validation were performed, and the results were pooled.

Within each outer training fold, the optimal number of PLS components was selected using an inner LOO procedure. The maximum number of components was capped at min (*n* = 2, *p* = 10). The selected model was then used to predict the observations held out in the outer fold. These observations were not used for either component selection or model fitting. Nested *Q*^2^ was calculated as described in Equation 4. PRESS represented the pooled prediction error sum of squares from the outer-held-out predictions, while SS_tot_ represented the total sum of squares of the observed trait values. Genuinely held-out predictions, rather than a refitted training model, form the basis of *Q*^2^; therefore, it accounts for model complexity. This makes *Q*^2^ suitable for comparing predictor categories with different numbers of features, ranging from one to 130, without requiring a separate complexity-matching procedure.

(4) $$Q^{2}=1-\frac{\text{PRESS}}{{\text{SS}}_{\text{tot}}}$$

##### 2.4.5.1 The permutation null model

The real *k*-means cluster assignments were compared with random partitions having the same cluster sizes. This test evaluated whether the observed clustering captured more trait information than expected by chance. For each predictor category, trait, site, and stage, 500 random permutations of the cluster labels were generated while preserving the original cluster sizes. The pooled leave-one-out *Q*^2^ was then calculated for each permuted partition. An empirical *p*-value was calculated as the proportion of permuted partitions with a pooled *Q*^2^ equal to or greater than that of the observed clustering.

##### 2.4.5.2 Hold-out-genotype validation

For each combination of predictor category, trait, site, and stage, genotypes were repeatedly divided into training and test sets using 30 random 75%/25% splits. *k*-means clustering (*k* = 3) and PLS models were fitted using only the training genotypes. The held-out genotypes were then assigned to the nearest training-cluster centroid based on Euclidean distance in the scaled feature space. Their trait values were predicted using the corresponding cluster-specific PLS model.

Hold-out *Q*^2^ was calculated by pooling squared prediction errors and total sums of squares across all held-out genotypes and repeats. The results were then compared with an equivalent procedure using random training-set partitions rather than *k*-means clusters. This approach directly tests whether clustering based on thermal features improves prediction for genotypes that were not used to define the clusters. It therefore addresses the potential inflation of within-cluster *R*^2^ caused by subsetting the dataset.

## 3 Results

### 3.1 Canopy temperature ranges across sites and stages

Across all phenological stages, *T*_c_ showed a clear daily pattern, with lower values recorded in the morning and an increase toward the afternoon (**Figure 2**). However, the degree of daily warming and variability among genotypes differed between environments and stages. Under WS, *T*_c_ rose more steeply during the afternoon hours than under FI across all stages, with MS producing the highest peak values, reaching a maximum early in the afternoon. This afternoon temperature was consistent with the MS stage coinciding with the warmest period of the Chilean summer at this dryland location. Under FI, daily *T*_c_ patterns were relatively more moderate, with lower peak values during ANT and MS. During the advanced reproductive stages (EGF and LGF), the trend reversed. Peak afternoon *T*_c_ values under FI (15:00–16:00 h) exceeded those at WS. At WS, canopy warming had already plateaued earlier in the afternoon by these stages (**Figure 2**).

**Figure 2:**
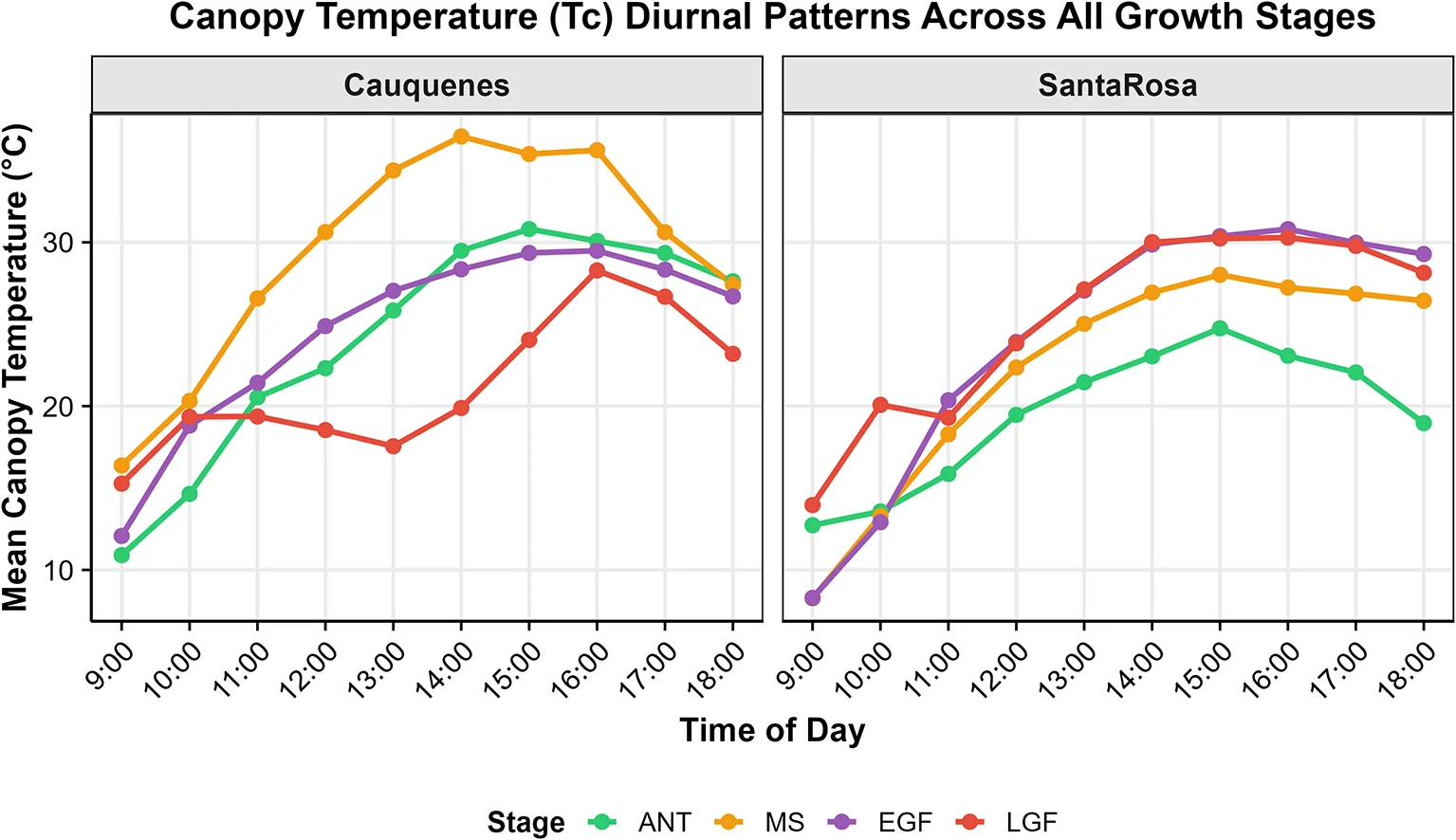
Canopy temperature (*T*_c_) diurnal patterns across all growth stages and sites. Mean hourly *T*_c_ (°C) for 196 genotypes at FI (left) and WS (right) across ANT, MS, EGF, and LGF.

CTD recorded the dynamic response of transpirational cooling throughout the day and differed between environments and stages (**Figure 3**). At both sites, all stages began with cooler canopies, i.e., negative CTD values, at 09:00 h. However, the timing of zero-crossing and the magnitude of afternoon warming diverged markedly between WS and FI. At WS, zero-crossing occurred early, between 10:00 and 11:00 h across most stages, after which CTD rose continuously through the afternoon. MS reached the highest positive peak at 16:00 h, while EGF and LGF followed closely at the same hour. ANT showed the most gradual rise, remaining the lowest positive stage throughout the afternoon at WS. At FI, genotypes reflected more sustained transpiration in the early morning, as zero-crossing was delayed, occurring between 11:00 and 13:00 h depending on the stage. Afternoon positive peaks at FI were lower than at WS and occurred later in the day, around 17:00 h. EGF at FI was the exception, starting the most negative at 09:00 h and recovering most steeply through the morning. The spread of individual genotype CTD trajectories around the site mean was broader under WS than under FI, particularly during EGF and LGF (**Figure 3**).

**Figure 3:**
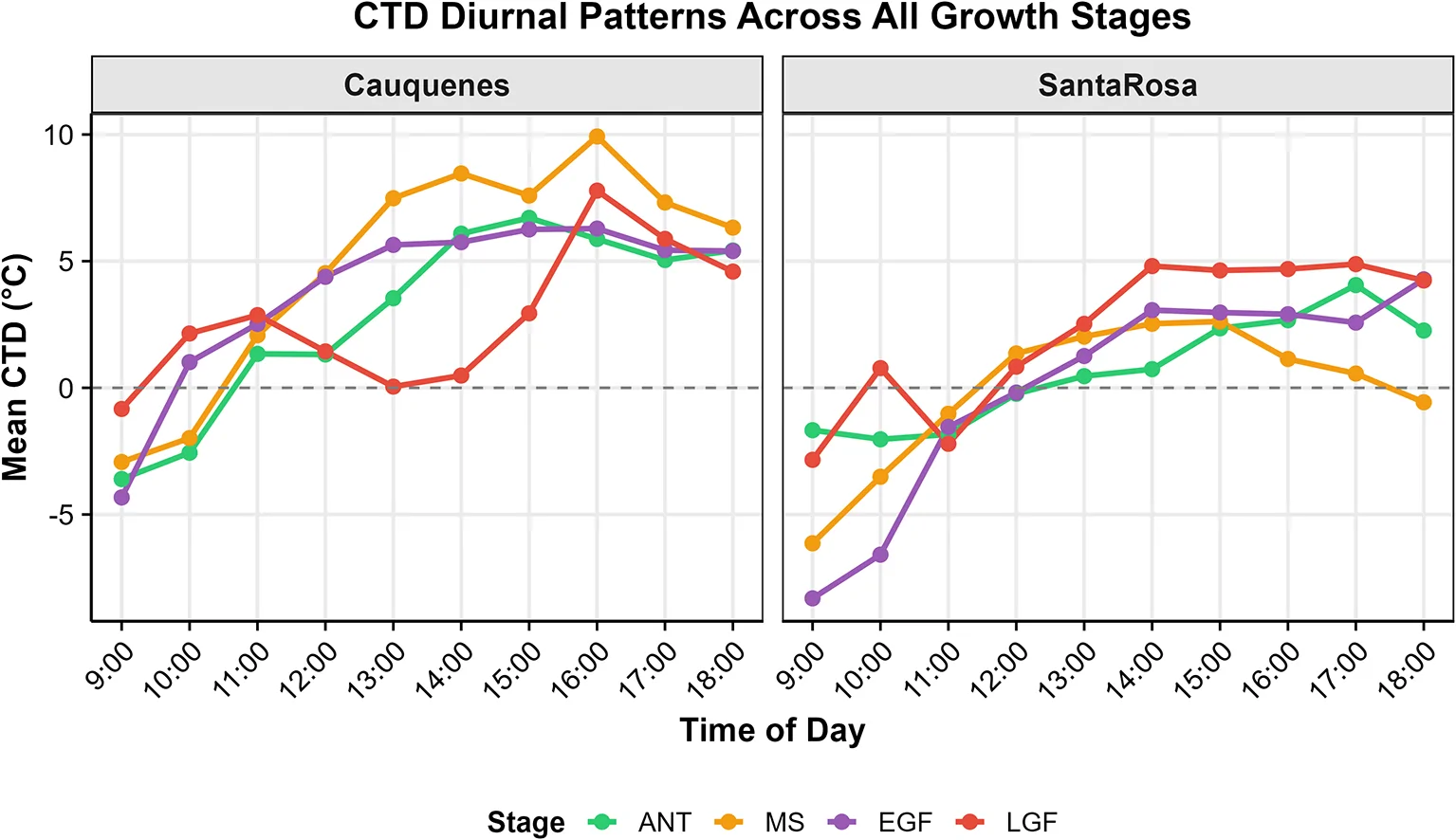
Canopy–air temperature differential (CTD) diurnal patterns across all growth stages and sites. Mean hourly CTD (°C) at FI (left) and WS (right) across ANT, MS, EGF, and LGF. Dashed line marks CTD = 0.

### 3.2 Diurnal curve shape diversity and BIC model selection

The best-fit diurnal model varied across sites and phenological stages, confirming that no single function adequately described all genotypes across both environments (**Figure 4**). At WS, *T*_c_ followed a clearly ordered seasonal progression through distinct parametric families from ANT to LGF, with each stage dominated by a different model. CTD at WS followed a separate sequence from *T*_c_ at the same stages.

**Figure 4:**
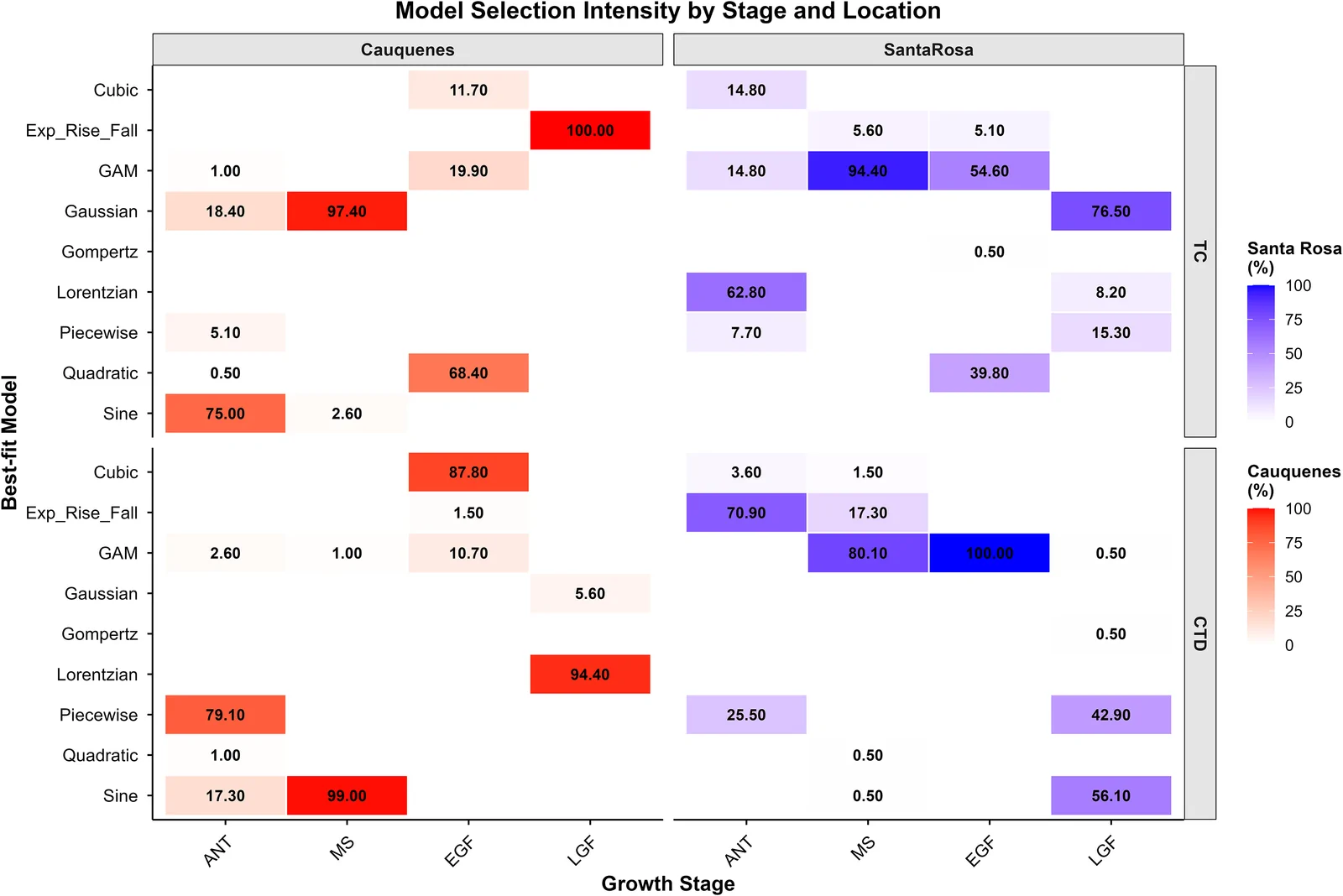
Model selection intensity by growth stage and site. Heatmap showing the proportion (%) of genotypes best fitted by each of 10 model families for *T*_c_ (top) and CTD (bottom) at FI (left) and WS (right) across ANT, MS, EGF, and LGF.

At FI, the pattern was fundamentally different. Rather than transitioning through different parametric families, FI relied heavily on nonparametric GAM during the intermediate stages. GAM dominated *T*_c_ at MS and was the sole best-fit model for CTD at EGF, before both *T*_c_ and CTD converged back toward parametric models at LGF. The contrast between WS and FI was most pronounced at MS and EGF, where WS maintained structured parametric dominance while FI was characterized by GAM throughout.

At LGF, both sites converged back toward single dominant parametric models, but on different families. WS selected asymmetric Exp_Rise_Fall for *T*_c_ and symmetric Lorentzian for CTD, while FI selected symmetric Gaussian for *T*_c_ and split between symmetric Sine and asymmetric Piecewise for CTD.

The best-fitting model was often different for *T*_c_ and CTD, even under the same site and phenological stage. At WS ANT, *T*_c_ was dominated by Sine, while CTD of the same genotypes was dominated by Piecewise. At FI ANT, *T*_c_ was dominated by Lorentzian, while CTD was dominated by Exp_Rise_Fall. This decoupling occurred consistently across sites and stages, indicating that *T*_c_ and CTD encode partly independent aspects of the diurnal thermal trajectory. Overall model fit quality was high at both sites, with a median *R*^2^ of 0.972 at WS and 0.977 at FI, and fewer than 0.1% of fits were nonsignificant (*p* ≥ 0.05).

### 3.3 Best predictive hour varies by stage and site

When each hourly *T*_c_ reading was assessed individually against the seven agronomic traits, the hour yielding the highest *R*^2^ shifted across phenological stages and sites with no consistent pattern (**Table 2**). At WS, the strongest single-hour predictor for GY occurred at 11:00 h during EGF (*R*^2^ = 0.145) but at 13:00 h during MS (*R*^2^ = 0.092) and at 09:00 h for several traits during LGF. At FI, the most predictive hour ranged from 09:00 h (GY and HI at MS) to 11:00 h (TKW, KS, KM2, and SM2 at EGF) to 15:00 h (SDW at MS, EGF, and LGF). Across both sites and all traits, the conventional 14:00 h reading was the best single hour in about 9% (five of 56) of stage-trait combinations.

**Table 2:** Best single-hour *T*_c_ predictor for each trait, site, and phenological stage.

Traits	Metric	WS (ANT)	WS (MS)	WS (EGF)	WS (LGF)	FI (ANT)	FI (MS)	FI (EGF)	FI (LGF)
GY	Best time	11:00	13:00	11:00	11:00	18:00	9:00	14:00	14:00
	R²	0.02	0.092	0.145	0.049	0.116	0.085	0.088	0.128
	14:00 (R²)	0.019	0.053	0.013	0.029	0.07	0.048	0.088	0.128
HI	Best time	17:00	9:00	18:00	18:00	17:00	9:00	14:00	13:00
	R²	0.043	0.016	0.021	0.01	0.026	0.017	0.018	0.027
	14:00 (R²)	0.00	0.001	0.006	0.008	0.001	0.002	0.018	0.022
TKW	Best time	14:00	9:00	11:00	9:00	14:00	11:00	11:00	10:00
	R²	0.009	0.01	0.002	0.004	0.057	0.123	0.184	0.194
	14:00 (R²)	0.009	0.001	0.001	0.00	0.057	0.066	0.11	0.154
KS	Best time	17:00	13:00	18:00	9:00	13:00	9:00	11:00	17:00
	R²	0.025	0.017	0.014	0.011	0.027	0.01	0.013	0.018
	14:00 (R²)	0.005	0.01	0.00	0.001	0.018	0.00	0.001	0.012
KM2	Best time	17:00	13:00	13:00	9:00	16:00	10:00	11:00	10:00
	R²	0.045	0.028	0.014	0.042	0.047	0.058	0.067	0.019
	14:00 (R²)	0.002	0.007	0.004	0.00	0.009	0.001	0.00	0.00
SDW	Best time	15:00	13:00	11:00	9:00	16:00	15:00	15:00	15:00
	R²	0.017	0.027	0.027	0.059	0.113	0.133	0.143	0.156
	14:00 (R²)	0.014	0.009	0.01	0.021	0.071	0.093	0.134	0.146
SM2	Best time	9:00	15:00	13:00	9:00	16:00	11:00	11:00	10:00
	R²	0.04	0.017	0.007	0.017	0.017	0.065	0.043	0.033
	14:00 (R²)	0.00	0.00	0.005	0.003	0.00	0.00	0.001	0.007

Each cell shows the hour of maximum *R*^2^ and its *R*^2^ value, alongside the conventional 14:00 h control *R*^2^. Bold hours differ from 14:00 h.

### 3.4 Multivariate diurnal features outperform the 14:00 h control

Across all seven agronomic traits, using all features within a predictor category in a PLS regression raised *R*^2^ above the 14:00 h control in the vast majority of site–stage combinations (**Figure 5**; **Supplementary Figures 1**-**6**, No_Clustering column). The largest multivariate gains were observed for traits that have near-zero *R*^2^ for the 14:00 h control. For example, SM2, GY, HI, and KS outperformed the control in over 90% of instances. The extent of improvement varied between the two sites. For yield-related traits, FI produced higher *R*^2^ values than WS across most stages. The difference was largest for TKW and KM2. For these traits, the improvement over the 14:00 h control was more than double at FI compared to WS. For biomass-related traits (SDW, SM2), both sites exhibited comparable gains during the early stages, with FI maintaining an advantage at MS and EGF. Among the predictor categories, raw hourly *T*_c_ and CTD readings demonstrated comparable performance to curve parameters for the majority of traits. However, ROC features proved to be particularly informative for SM2 and KM2.

**Figure 5:**
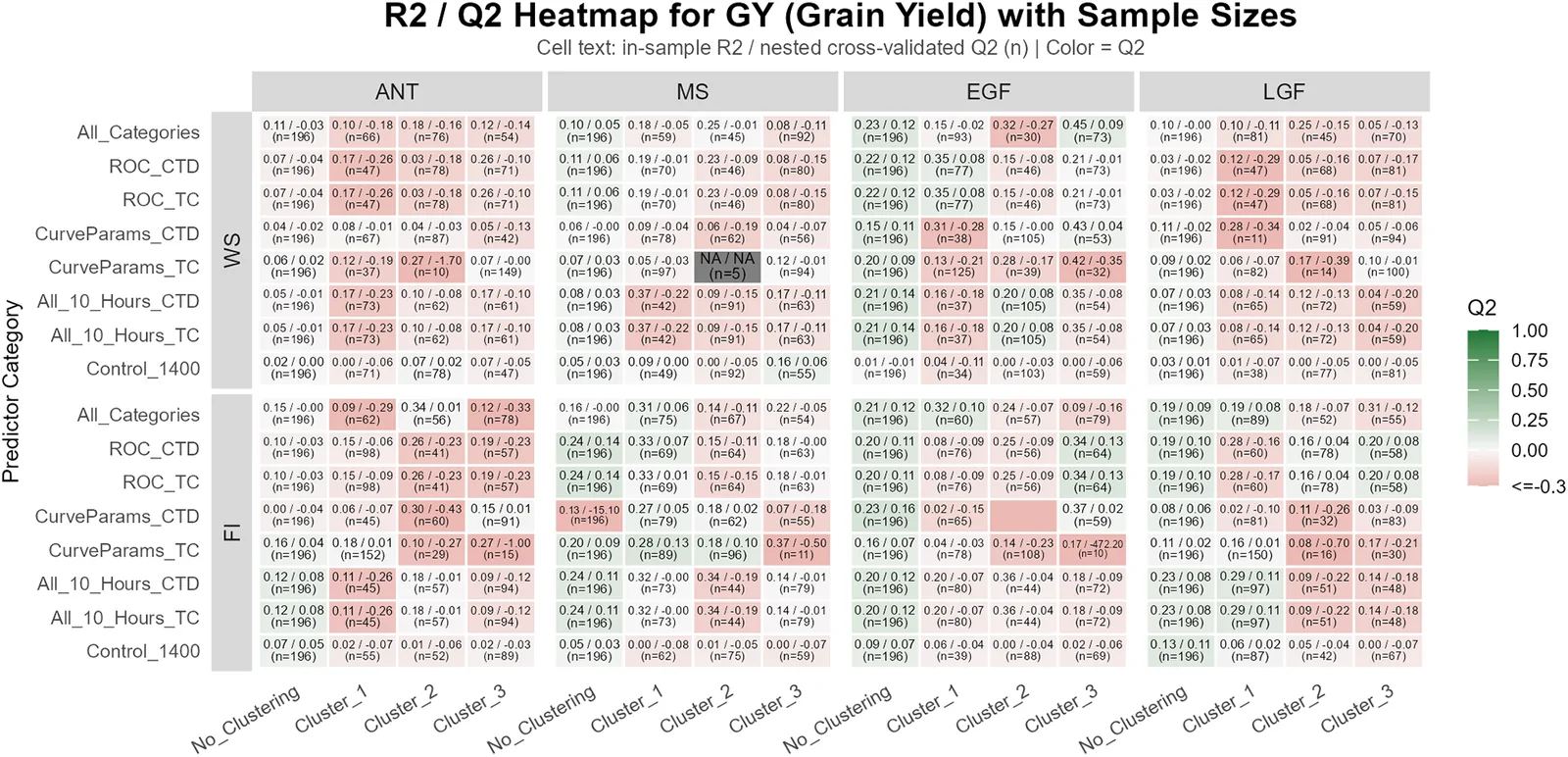
Within-cluster nested cross-validated *Q*^2^ (in-sample *R*^2^ shown in parentheses) for grain yield (GY) across predictor categories and phenological stages at WS and FI, derived from partial least squares (PLS) regression fitted within each k-means cluster (*k* = 3). Each cell shows the *Q*^2^ value, the in-sample *R*^2^ in parentheses, and the number of genotypes in that cluster (*n*). Predictor categories range from one feature (Control_1400, the single 14:00 h *T*_c_ reading) to 130 features (All_Categories).

This in-sample advantage did not persist under cross-validation. Median nested *Q*^2^ for the no-clustering, whole-panel PLS models was at or below zero for all eight predictor categories. This included the 14:00 h control, which had a median *Q*^2^ of − 0.014. The other categories ranged from − 0.009 to − 0.023. These results were obtained when genuinely out-of-sample predictions were used instead of training set refits; none of the predictor categories showed a reliable whole-panel predictive advantage over the single-hour control. This was also true for the multivariate categories containing five to 130 features. Therefore, at the current sample size (*n* = 196), the in-sample *R*^2^ differences reported above and shown in **Figure 5**; **Supplementary Figures 1–6** (No-Clustering column) should be interpreted as an upper bound on model performance rather than evidence of a validated predictive advantage.

### 3.5 Genotypic clustering: cross-validated assessment

Applying *k*-means clustering (*k* = 3) to the diurnal thermal feature space raised within-cluster *R*^2^ above the No_Clustering baseline for at least one cluster in every trait–stage–site combination tested (**Figure 5**; **Supplementary Figures 1**–**6**), with individual clusters reaching *R*^2^ as high as 0.85. However, several clusters contained only 10–15 genotypes while being fitted with five to 130 predictors. Therefore, these in-sample *R*^2^ values were reevaluated using nested cross-validated *Q*^2^, a permutation-based null model, and hold-out-genotype validation.

Across the full set of 448 category × trait × site × stage combinations, 1,333 cluster-level cells were evaluated after excluding the no-clustering baseline. Only 10.6% of these cells had positive nested *Q*^2^ values. Moreover, just 1.6% exceeded a *Q*^2^ of 0.10. Median *Q*^2^ was negative across all eight predictor categories. The All-Categories predictor set contained 130 features but did not outperform the simpler categories when evaluated using *Q*^2^. Its median *Q*^2^ was -0.132, and only 10.7% of cells had positive *Q*^2^. Both values were among the lowest across the eight predictor categories. These results indicate that the additional predictors in the combined features set did not provide meaningful predictive information when evaluated out of sample.

The nine cluster-trait-stage-site combinations with the strongest in-sample *R*^2^ (**Table 3**) showed a similar pattern under validation. Five of the nine had zero or negative nested *Q*^2^ values. The remaining four showed positive *Q*^2^ in isolation but did not pass the permutation test, the hold-out-genotype test, or both. Only one combination, kernels per m^2^ (KM2) predicted from CurveParams_TC under WS EGF (*n* = 39), showed both positive nested *Q*^2^ (0.11) and a significant permutation result (*p* = 0.002). However, this combination performed substantially worse than a random partition of the same size when evaluated on held-out genotypes. Hold-out *Q*^2^ was − 0.73 for the k-means clusters compared with − 0.10 for the random partitions. Thus, even this apparently strong result did not generalize to new genotypes. Across all nine combinations, no cluster-based partition outperformed a size-matched random partition when evaluated on held-out genotypes (**Table 3**).

**Table 3:** Validated reassessment of the nine cluster-trait-stage-site combinations having the best *R*^2^.

Trait, category, site stage	Original *R*^2^ (cluster *n*)	Nested *Q*^2^	Permutation *p*	Hold-out *Q*^2^: *k*-means vs. random	Validation reference
HI, CurveParams_TC, FI MS	0.84 (*n* = 11)	− 0.31	0.038	− 0.054 vs. − 0.039	**Supplementary Figure 1**
TKW, CurveParams_TC, FI ANT	0.82 (*n* = 15)	0.075	0.082 (n.s.)	− 0.082 vs. − 0.072	**Supplementary Figure 2**
TKW, CurveParams_TC, WS LGF	0.65 (*n* = 14)	0.272	0.028*	− 0.038 vs. − 0.043	**Supplementary Figure 2**
KM2, CurveParams_TC, WS EGF	0.53 (*n* = 39)	0.113	0.002	− 0.732 vs. − 0.096	**Supplementary Figure 4**
KM2, CurveParams_TC, FI EGF	0.57 (*n* = 10)	0.327	0.136 (n.s.)	− 0.121 vs. − 0.103	**Supplementary Figure 4**
SDW, All_Categories, WS EGF	0.77 (*n* = 30)	− 0.110	0.206 (n.s.)	− 0.067 vs. − 0.159	**Supplementary Figure 5**
SDW, ROC_TC, FI ANT	0.68 (*n* = 41)	− 0.207	0.228 (n.s.)	− 0.083 vs. − 0.108	**Supplementary Figure 5**
SM2, CurveParams_TC, FI EGF	0.85 (*n* = 10)	0.757	0.160 (n.s.)	− 0.044 vs. − 0.295	**Supplementary Figure 6**
SM2, ROC_TC, WS EGF	0.64 (*n* = 46)	0.068	0.433 (n.s.)	− 0.154 vs. − 0.155	**Supplementary Figure 6**

These results indicate that the apparent within-cluster *R*^2^ improvements reported for individual clusters in this study (**Figure 5**; **Supplementary Figures 1**–**6**) were, in most cases, likely driven by *R*^2^ inflation in small, high-dimensional samples. They therefore do not provide evidence of a genotypic stratification effect that generalizes beyond the genotypes used to fit each model. The physiological hypothesis underlying this analysis remains plausible. Contrasting thermo-hydraulic strategies could potentially produce biologically coherent thermal subgroups within the panel. However, such effects may be difficult to detect reliably when clusters contain only 10–77 genotypes and are modeled using dozens to more than 100 correlated predictors. Nevertheless, the present dataset does not provide validated statistical support for this hypothesis at the current panel size. Therefore, the specific cluster-based predictions should not be interpreted as validated findings.

The strongest in-sample association for GY was obtained with the full combined feature set (All_Categories) in a 73-genotype cluster at WS EGF (*R*^2^ = 0.45). This cell also retained a modestly positive nested *Q*^2^ (0.092) (**Figure 5**). It was one of only 32 of 192 GY cluster cells (17%) with positive *Q*^2^. No GY cluster reached the magnitude observed for several other traits.

The largest in-sample gain for HI was obtained with CurveParams_TC at FI MS (*R*^2^ = 0.84, *n* = 11; **Table 3**). However, nested *Q*^2^ was strongly negative (− 0.31) (**Supplementary Figure 1**). The cluster also performed worse than a random partition of the same size on held-out genotypes. Across the full HI grid, only 6% of cluster cells retained positive *Q*^2^. This was the lowest proportion among the seven traits and represented the weakest validated signal.

CurveParams_TC at FI ANT produced a high in-sample *R*^2^ (0.82, *n* = 15; **Table 3**). However, nested *Q*^2^ was only 0.075 and was not significant under permutation testing (*p* = 0.082). CurveParams_TC at WS LGF showed a higher nested *Q*^2^ (0.272; *R*^2^ = 0.65, *n* = 14). However, the permutation test for this cell used a simpler leave-one-out statistic rather than nested *Q*^2^. Therefore, its associated p-value (0.028) should not be interpreted as confirmation of the nested-*Q*^2^ result. On held-out genotypes, the cluster performed no better than a random partition of the same size (*Q*^2^ = − 0.038 vs. − 0.043). Thus, the association did not generalize to unseen genotypes. Across the TKW grid, 11% of cluster cells retained positive *Q*^2^ (**Supplementary Figure 2**).

The strongest KS association was obtained with CurveParams_TC at FI EGF (*R*^2^ = 0.60, *n* = 10). This was also the best-validated cell for KS, with a nested *Q*^2^ of 0.186. Overall, only 7% of KS cluster cells retained positive *Q*^2^ (**Supplementary Figure 3**).

CurveParams_TC at WS EGF produced an in-sample *R*^2^ of 0.53 in a 39-genotype cluster (**Table 3**). The nested *Q*^2^ was positive (0.113), and this was the only combination showing a clearly significant permutation result (*p* = 0.002). However, its performance on held-out genotypes was substantially worse than that of a random partition (*Q*^2^ = − 0.73 vs. − 0.10). CurveParams_TC at FI EGF produced a higher nested *Q*^2^ (0.327; *R*^2^ = 0.57, *n* = 10), but the permutation test was not significant (*p* = 0.136) (**Supplementary Figure 4**). Overall, only 8% of KM2 cluster cells retained positive *Q*^2^.

All_Categories at WS EGF produced an in-sample *R*^2^ of 0.77 in a 30-genotype cluster (**Table 3**). However, nested *Q*^2^ was negative (− 0.110). ROC_TC at FI ANT also showed a high in-sample *R*^2^ (0.68, *n* = 41) but a negative nested *Q*^2^ (− 0.207). The permutation test was not significant (*p* = 0.228). Separately, the highest nested *Q*^2^ in the SDW grid was obtained with ROC_CTD at WS EGF (*R*^2^ = 0.55, *n* = 46; *Q*^2^ = 0.291) (**Supplementary Figure 5**). This result was more promising on the *Q*^2^ metric than the two SDW combinations described above.

CurveParams_TC at FI EGF produced the highest in-sample *R*^2^ (0.85, *n* = 10; **Table 3**). It also retained the highest nested *Q*^2^ in the study (0.757). However, the permutation test was not significant (*p* = 0.160). ROC_TC at WS EGF produced a smaller positive nested *Q*^2^ (0.068; *R*^2^ = 0.64, *n* = 46), which was also not significant (*p* = 0.433). On held-out genotypes, its performance was essentially identical to that of a random partition (*Q*^2^ = − 0.154 vs. − 0.155) (**Supplementary Figure 6**).

## 4 Discussion

### 4.1 One snapshot underestimates diurnal thermal information

A single 14:00 h *T*_c_ reading, while operationally convenient, treats canopy temperature as a static trait and implicitly assumes that the mid-afternoon thermal state represents the genotype’s full cooling capacity. This assumption has been challenged repeatedly in the literature (DeJonge et al., 2015). Repeated-measurement studies in wheat have shown that the relationship between canopy temperature and yield varies with measurement time and atmospheric conditions, and that a single observation is an unreliable proxy for the genotype’s thermal behavior across the day (Balota et al., 2007; Flores-Olave et al., 2026; Thapa et al., 2018). The present study confirms and extends this concern as the most predictive measurement hour was inconsistent across traits, stages, and sites, and the 14:00 h reading was rarely optimal (**Table 2**). When all features within a category were used jointly in a PLS regression, in-sample trait associations improved over the single-time control across the large majority of site–stage combinations for all seven traits. However, this in-sample comparison did not hold up under cross-validated *Q*^2^. This pattern is independent of the validation approach and remains informative. No single fixed measurement hour consistently captured the trait-relevant thermal signal across all conditions. Prior work has argued that time-integrated thermal information outperforms a single mid-afternoon reading (Flores-Olave et al., 2026; Thapa et al., 2018); the present results support the weaker, hour-inconsistency version of that argument (**Table 2**), but once cross-validated, they do not support a validated multivariate predictive advantage over the single-hour control at this panel size. It also aligns with findings from multitemporal UAV campaigns showing that the optimal genotypic discrimination window shifts with phenological stage and environmental conditions (Qin et al., 2022; Treier et al., 2025).

The contrasting patterns of category dominance between sites carry physiological meaning. At WS, ROC features were frequently the most informative category, particularly for GY, SDW, and SM2. The rate of morning canopy warming is a direct indicator of stomatal behavior under rising VPD (Grossiord et al., 2020). Genotypes whose canopy heats rapidly in the early hours have already restricted transpiration before peak atmospheric demand arrives. This limits carbon assimilation during the most productive window of the day (Koehler et al., 2024; Schoppach and Sadok, 2012). Under dryland conditions where soil water is progressively limiting, this stomatal strategy diverges sharply among genotypes by mid-morning, making early-day thermal dynamics particularly discriminating (Moritz et al., 2024; Romero-Bravo et al., 2019). At FI, where sustained water availability maintains transpirational cooling well into the afternoon, CurveParams_TC emerged as the more consistently informative category. Here, the integrated shape of the diurnal *T*_c_ trajectory, including its peak timing, asymmetry between morning warming and afternoon cooling, and total thermal load, carries the genotypic signal because differences in stomatal strategy are expressed across the full day rather than concentrated in the morning warming phase (Flores-Olave et al., 2026; Ma et al., 2021). Traits with the weakest single-time-point associations, including HI and SM2, benefited most from the multivariate approach in relative terms, suggesting that their physiological determinants are distributed across the full diurnal cycle rather than concentrated at any one measurement hour (Perich et al., 2020). The similar performance observed between ROC_TC and ROC_CTD, and between All_10_Hours_TC and All_10_Hours_CTD, is expected rather than coincidental. CTD = *T*_c_ − *T*_a_. *T*_a_ is common across genotypes at a given hour and site–stage combination; therefore, CTD differs from *T*_c_ by a constant, genotype-invariant offset at that hour. The rate of change in CTD across hours is not necessarily equivalent to that of *T*_c_ because *T*_a_ also varies across hours. As a result, features derived from these two variables may capture overlapping but not identical information. These physiological interpretations of predictor-category dominance are based on in-sample patterns. As noted above and in the Results, they were not supported by cross-validated *Q*^2^. Therefore, they should be considered hypotheses regarding the physiological basis of differences among predictor categories rather than validated findings.

### 4.2 Diurnal curve shape reflects site-specific thermal dynamics

The BIC-based model selection showed that the shape of the diurnal *T*_c_ trajectory changes progressively with phenological stages. It follows a sequence from symmetric Sine curves at ANT to Gaussian peaks at MS to Exponential Rise-Fall at LGF. This progression mirrors the seasonal evolution of atmospheric demand and canopy physiology at this dryland site (Flores-Olave et al., 2026). The near-universal dominance of Gaussian models at MS reflects the well-defined midday peak typical of a clear semi-arid day with high but symmetric evaporative demand. The shift to Exp_Rise_Fall at LGF reflects an asymmetric diurnal pattern where the canopy warms sharply in the morning but cools slowly in the afternoon. This is consistent with progressive senescence reducing transpirational cooling capacity and stomatal conductance during the grain-filling period (Narayanan, 2018; Faralli et al., 2019; Ma et al., 2021). The optimal models for *T*_c_ and CTD frequently decoupled within the same environment and stage, such as following a Sine curve for *T*_c_ but a Piecewise model for CTD at WS ANT. This divergence indicates that absolute temperature and the canopy–air differential encode partly independent information about the genotype’s thermal status (Chai et al., 2022). Therefore, *T*_c_ and CTD should be treated as separate feature sources rather than interchangeable proxies (Neukam et al., 2016).

At FI, the widespread dominance of GAM at MS and EGF indicates that no fixed parametric shape adequately captures the diurnal trajectories generated by sustained irrigation. Irrigation modifies field microclimate by sustaining transpirational cooling, attenuating afternoon VPD peaks, and creating canopy-level temperature feedbacks that deviate from standard bell-shaped or exponential patterns (Luan and Vico, 2021). The complete dominance of GAM for CTD at FI EGF points specifically to complex, nonsymmetric patterns in the canopy–air differential at this stage, likely reflecting genotypic heterogeneity in the timing and magnitude of stomatal closure that no single parametric function can capture (van Voorn et al., 2023). The high overall model fit quality at both sites confirms that the ten-model candidate set was adequate for the range of curve shapes present in the data and that BIC penalization effectively prevented overfitting while allowing flexible models to win when the data required them (Schwarz, 1978).

### 4.3 Genotypic stratification by diurnal thermal profile: what the validated evidence shows

In-sample within-cluster *R*^2^ exceeded the no-clustering baseline for at least one cluster across every trait, growth stage, and site combination tested. In contrast, the 14:00 h control showed no comparable in-sample improvement under any condition (**Figure 5**; **Supplementary Figures 1**-**6**). Taken at face value, this pattern suggests that the diurnal thermal feature space contains biological structure that may stratify genotypes into subgroups with stronger trait associations. However, formal testing against a permutation null model and genuinely held-out genotypes did not support this interpretation for the combinations examined (see Results and Cross-validated model assessment). Cluster-based partitions did not outperform size-matched random partitions on held-out genotypes for any of the nine combinations tested. This included the single combination that showed a significant result in the in-sample permutation test. These results do not demonstrate that such biological structure is absent. Clusters containing 10–77 genotypes and fitted with 5–130 correlated predictors may have limited power to detect real but modest effects (Wold et al., 2001). The physiological rationale for contrasting water-use strategies within the panel therefore remains a plausible hypothesis. However, the present dataset does not provide validated evidence for the proposed stratification. Accordingly, the specific cluster-based associations should not be considered established findings. A plausible physiological explanation for the in-sample pattern, though not validated by this study, involves contrasting water-use strategies within the panel (Qin et al., 2022). Transpiration-efficient genotypes sustain stomatal aperture and canopy cooling throughout the day under increasing VPD, while water-conservative genotypes restrict transpiration earlier and accept a warmer afternoon canopy in exchange for preserving soil moisture for later grain filling (Estrada et al., 2023; Schoppach and Sadok, 2012; Sofi et al., 2019). When pooled, these strategies could produce conflicting temperature–trait signals that weaken any regression fit; clustering on the full diurnal profile is intended to separate them, which would in principle make within-group relationships stronger and more physiologically coherent (Balota et al., 2007). If this mechanism holds, the site-specific pattern of which feature category produced the largest in-sample gain would be consistent with it, although neither pattern has been confirmed by the validation analyses above.

Under WS, the rate of early morning warming (ROC features) served as the primary discriminating signal in-sample. Conversely, under FI, the integrated curve shape (CurveParams_TC) captured apparent genotypic differences. This matches findings from clustering of diurnal *T*_c_ profiles in wheat, where group membership was determined by differences in cooling patterns rather than absolute temperature levels (Flores-Olave et al., 2026). However, the separation between these groups in this study did not validate against held-out genotypes, so this parallel should be read as motivation for further work rather than as corroboration. Furthermore, the separation between these groups was more distinct under irrigated conditions than under rainfed conditions in prior work (Flores-Olave et al., 2026).

### 4.4 Practical implications for wheat breeding programs

The inconsistency of the best predictive hour across traits, stages, and environments has direct practical consequences for thermal phenotyping protocols. Programs relying on a single fixed afternoon measurement cannot be confident that they are capturing the most informative thermal signal for any given combination of site, stage, and target trait. This is particularly consequential under dryland conditions, where within-day genotype reranking increases sharply as atmospheric demand rises through the morning, meaning that the relative performance of genotypes at 14:00 h may differ from their ranking at the more informative earlier hours (Flores-Olave et al., 2026; Balota et al., 2007). Adding a small number of additional hourly readings during the morning warming phase, which modern thermal UAV platforms can acquire at negligible additional cost per flight, would substantially expand the descriptive value of existing phenotyping campaigns without requiring changes to equipment or experimental design (Perich et al., 2020; Gano et al., 2024).

Cluster-based stratification using the full diurnal thermal phenotype remains a conceptually promising complement to single-hour phenotyping. However, the validated results of this study do not yet support its use in breeding programs. The clusters evaluated here did not outperform random genotype groupings when tested on held-out genotypes. Before this approach can be adopted operationally, it should be validated using an independent genotype panel or growing season. Such validation should include nested cross-validation, permutation testing, and hold-out-genotype testing, as applied in the present study. Larger cluster sizes than the 10–77 genotypes available in this single-panel dataset would also provide greater statistical power.

Future work should extend the diurnal feature framework to multiyear datasets to assess the repeatability of cluster assignments, integrate thermal phenotypes with genomic and multitrait selection models, and evaluate whether the identified ROC and CurveParams features map to quantitative trait loci associated with stomatal conductance and transpiration efficiency under Mediterranean drought-heat conditions (Anderegg et al., 2024; Riaz et al., 2021; Rutkoski et al., 2016). Alignment with such genomic regions is noted here as a direction for future work and is not addressed by the present dataset.

## 5 Limitations

Each phenological stage at each site was measured using hourly thermal imaging on a single day. Diurnal CT and CTD trajectories are strongly influenced by daily weather conditions, including cloud cover, wind speed, and vapor pressure deficit. Therefore, the curve features extracted in this study cannot be fully separated from the atmospheric conditions specific to each measurement day. This limitation is more fundamental than the need for multiyear repeatability testing discussed below. Genotypic effects and single-day weather effects are structurally confounded within each stage-level curve fit. Consequently, these effects cannot be disentangled using the present single-day-per-stage design.

Within-cluster *R*^2^ and *Q*^2^ estimates from the smallest clusters, which contained as few as 10–15 genotypes and included up to 130 predictors, should be interpreted cautiously. These estimates remain susceptible to high sampling variability, even when cross-validation is applied. PLS component selection can reduce model complexity but cannot fully eliminate small-sample instability when cluster size approaches the number of predictors. Independent validation across growing seasons and environments is therefore needed to determine whether genotype–thermal-profile associations are repeatable. Future studies should use genotype panels and thermal feature sets defined in advance before such associations are considered for cluster-based selection decisions in breeding programs (Anderegg et al., 2024; Rebetzke et al., 2012; Riaz et al., 2021).

## 6 Conclusion

The conventional 14:00 h *T*_c_ reading was the most predictive single measurement time in less than 10% of stage–trait–site combinations tested, with the most informative hour shifting unpredictably across traits, phenological stages, and environments. This inconsistency alone demonstrates that no fixed measurement time reliably captures the trait-relevant thermal signal across diverse breeding conditions.

When all features within each predictor category were used in a PLS regression across all 196 genotypes, in-sample *R*^2^ exceeded the 14:00 h control for most site–stage combinations for all seven agronomic traits. *k*-means clustering of the diurnal feature space further increased within-cluster *R*^2^, reaching up to 0.85 in some clusters. However, these apparent gains did not persist under cross-validation. Cross-validated *Q*^2^, permutation testing, and hold-out-genotype validation indicated that the observed improvements were largely attributable to *R*^2^ inflation in small, high-dimensional samples rather than to genotypic stratification that generalized to unseen genotypes. At the whole-panel level, median cross-validated *Q*^2^ was near zero or negative for every predictor category. Moreover, none of the *k*-means clusters outperformed size-matched random genotype partitions when evaluated on held-out genotypes. The All_Categories predictor set, which contained the largest number of features (130 predictors), also did not outperform the simpler categories after accounting for model complexity using *Q*^2^. Thus, a larger feature set did not provide a validated predictive advantage. For the specific cluster–trait combinations examined in detail, validated *Q*^2^ values ranged from − 0.31 to 0.76 (**Table 3**). These values provide quantitative support for the observed variation in predictive performance rather than relying on qualitative descriptions.

Taken together, these results support two main conclusions. First, diurnal thermal phenotyping provides more information than a single fixed measurement time for identifying trait-relevant hours and feature types. Therefore, no single measurement hour or feature category should be assumed to be optimal in advance. Second, and more importantly, in-sample *R*^2^ substantially overestimated the value of genotypic stratification based on k-means clustering of diurnal thermal features at the panel size used here. The specific clustering-based predictions identified in this study should therefore not be considered validated. Cross-validated *Q*^2^, rather than in-sample *R*^2^, should be used as the primary metric for evaluating such claims in future studies.

Adding hourly *T*_c_ readings during the morning warming phase substantially expands the descriptive value of existing UAV-based thermal campaigns. No changes to equipment or flight protocols are required. Applying genotypic stratification based on diurnal thermal profiles to breeding decisions is not yet supported by validated evidence from this study. Further confirmation is needed using an independent genotype panel or growing season. Such validation should include nested cross-validation, permutation testing, and hold-out-genotype validation before this approach can be recommended for operational use. Future work should validate the identified thermal phenotype groups across growing seasons and environments. Their heritability and stability as selection criteria remain to be assessed. Alignment of these groups with genomic regions associated with stomatal conductance and transpiration efficiency under Mediterranean drought–heat conditions would further strengthen their utility in marker-assisted breeding.

## Data Availability

The original contributions presented in the study are included in the article/**Supplementary Material**. Further inquiries can be directed to the corresponding author.

## Supplementary material

Supplementary Figure S1 Within-cluster nested cross-validated Q² (in-sample R² shown in parentheses) for Harvest Index (HI) across predictor categories and phenological stages at WS and FI, derived from partial least squares (PLS) regression fitted within each k-means cluster (k = 3). Each cell shows the Q² value, the in-sample R² in parentheses, and the number of genotypes in that cluster (n). Predictor categories range from 1 feature (Control_1400, the single 14:00 h Tc reading) to up to 130 features (All_Categories).

Supplementary Figure S2 Within-cluster nested cross-validated Q² (in-sample R² shown in parentheses) for Thousand Kernel Weight (TKW) across predictor categories and phenological stages at WS and FI, derived from partial least squares (PLS) regression fitted within each k-means cluster (k = 3). Each cell shows the Q² value, the in-sample R² in parentheses, and the number of genotypes in that cluster (n). Predictor categories range from 1 feature (Control_1400, the single 14:00 h Tc reading) to up to 130 features (All_Categories).

Supplementary Figure S3 Within-cluster nested cross-validated Q² (in-sample R² shown in parentheses) for Kernels per Spike (KS) across predictor categories and phenological stages at WS and FI, derived from partial least squares (PLS) regression fitted within each k-means cluster (k = 3). Each cell shows the Q² value, the in-sample R² in parentheses, and the number of genotypes in that cluster (n). Predictor categories range from 1 feature (Control_1400, the single 14:00 h Tc reading) to up to 130 features (All_Categories).

Supplementary Figure S4 Within-cluster nested cross-validated Q² (in-sample R² shown in parentheses) for Kernels per m² (KM2) across predictor categories and phenological stages at WS and FI, derived from partial least squares (PLS) regression fitted within each k-means cluster (k = 3). Each cell shows the Q² value, the in-sample R² in parentheses, and the number of genotypes in that cluster (n). Predictor categories range from 1 feature (Control_1400, the single 14:00 h Tc reading) to up to 130 features (All_Categories).

Supplementary Figure S5 Within-cluster nested cross-validated Q² (in-sample R² shown in parentheses) for Shoot Dry Weight (SDW) across predictor categories and phenological stages at WS and FI, derived from partial least squares (PLS) regression fitted within each k-means cluster (k = 3). Each cell shows the Q² value, the in-sample R² in parentheses, and the number of genotypes in that cluster (n). Predictor categories range from 1 feature (Control_1400, the single 14:00 h Tc reading) to up to 130 features (All_Categories).

Supplementary Figure S6 Within-cluster nested cross-validated Q² (in-sample R² shown in parentheses) for Spikelets per m² (SM2) across predictor categories and phenological stages at WS and FI, derived from partial least squares (PLS) regression fitted within each k-means cluster (k = 3). Each cell shows the Q² value, the in-sample R² in parentheses, and the number of genotypes in that cluster (n). Predictor categories range from 1 feature (Control_1400, the single 14:00 h Tc reading) to up to 130 features (All_Categories).
