# Correction: Turning canopy temperature into a multidimensional tool: phenotyping diurnal canopy temperature dynamics in spring wheat breeding programs

**DOI:** 10.3389/fpls.2026.2023622

**Published:** 2026-10-20

**Authors:** Frontiers Production Office

**Affiliations:** Frontiers Media SA, Lausanne, Switzerland

**Keywords:** curve fitting, diurnal variation, K-Means clustering, Mediterranean environment, stomatal conductance, thermometry, yield components

The tables were in the wrong order in the published version of this paper. **Table 1** has been corrected to **Table 3**, **Table 2** has been corrected to **Table 1**, and **Table 3** has been corrected to **Table 2**. The order has now been corrected. All citations are correct as published.

The original version of this article has been updated.

